# Chiari I malformation presenting with ganglion cell complex thinning on routine examination

**DOI:** 10.5935/0004-2749.2022-0150

**Published:** 2025-08-21

**Authors:** Özkan Kocamış, Kemal Örnek, Emine Temel

**Affiliations:** 1 Department of Ophthalmolgy, Medicine Faculty, Ahi Evran University, Kırşehir, Turkey; 2 Department of Ophthalmolgy, Ahi Evran Education and Research Hospital, Kırşehir, Turkey

Dear Editor,

Chiari I malformation (CMI) is a rare congenital disorder characterized by the caudal displacement of cerebellar tonsils through the foramen magnum into the cervical canal^(1)^. Ophthalmological signs include retro-orbital pain, diplopia, photophobia, impaired visual acuity, nystagmus, strabismus, and papilledema^(2-4)^. The diagnosis is mostly based on magnetic resonance imaging (MRI) findings.

A 44-year-old female patient was admitted with a complaint of blurred vision in the left eye. She had no history of ophthalmological or neurological diseases. She had medically controlled diabetes mellitus for 5 years. She never drank alcohol or smoked cigarette. Her family history was unremarkable.

On examination, the best-corrected visual acuity was 10/10 in the right eye and 8/10 in the left eye. Ocular movements were painless and full in all directions with normal ocular alignment. The pupils were equal in size and reactive to light, and there were no relative afferent pupillary defects. The intraocular pressure was 15 mmHg on the right and 17 mmHg on the left eye by Goldmann applanation tonometry. The central corneal thickness was 571 µm in the right eye and 586 µm in the left eye. Examination of the anterior segment and fundus revealed a clear media and normal peripheral retina, macula, and optic disks. There were no neuroretinal rim defects and disk hemorrhages. The Ishihara color vision test on both eyes was normal.

**Figure 1:**
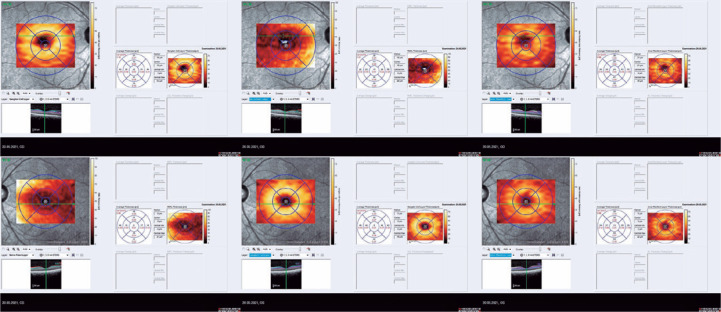
Segmentation analysis revealed decreased retinal GCC thickness in the left eye compared with that in the right eye (61 _µ_m versus 30 _µ_m, respectively).

On optical coherence tomography (OCT), the central macular thickness was decreased in the left eye compared with that in the right eye (270 µm versus 230 µm, respectively). The mean retinal nerve fiber layer (RNFL) thickness was comparable on both eyes (118 µm versus 111 µm). Segmentation analysis revealed decreased retinal ganglion cell complex (GCC) thickness in the left eye compared with that in the right eye (61 µm versus 30 µm) (Figure 1). Inter-eye asymmetry exceeding the normal limits suggests pathology.

**Figure 2:**
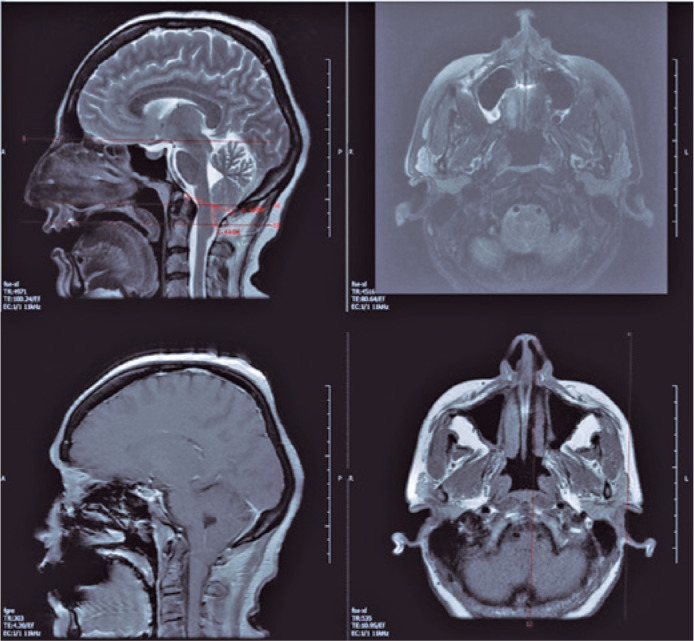
Brain magnetic resonance imaging with contrast; images of sagittal and axial T2 unenhanced series (top) and T1 sagittal and axial T1 contrast-enhanced series (bottom). The cerebellar tonsils herniate approximately 11 mm from the foramen magnum to the inferior. The lateral ventricles and fourth ventricle had normal width.

Brain MRI (Figure 2) revealed herniated cerebellar tonsils, approximately 11 mm from the foramen magnum to the inferior. The condition was not accompanied by hydrocephalus, space-occupying lesions, and cerebral venous thromboses. The finding was consistent with CMI.

The primary pathology in CMI is attributed to the obstruction of the cerebrospinal fluid (CSF) flow, more than the location of tonsillar descent below the foramen magnum. A structural abnormality in the posterior fossa can lead to severe increases in the CSF, and such an increase can present with acute visual loss and papilledema.

The patient did not have any symptoms of increased intracranial pressure. There were no clinical signs of optic nerve involvement, papilledema, or diabetic retinopathy in both eyes. Interestingly, our patient was initially diagnosed after the visualization of unilateral retinal GCC thinning on OCT.

Figus et al.^(5)^ evaluated OCT images of the optic nerve head in patients with CMI and measured the mean peripapillary RNFL thickness. They found decreased RNFL thickness in patients with CMI when compared with healthy controls; however, the decrease was more prominent in patients with syringomyelia and in those who underwent surgery.

In summary, correct and timely diagnosis of neurophthalmolgical conditions is vital to avoid unnecessary treatment of an optic neuropathy and a late diagnosis of a life-threatening intracranial pathology. OCT in patients with CMI can be a noninvasive imaging technique for the collection of information needed for diagnosis. Moreover, this technique may be useful for the monitoring of the RNFL, GCC damage, and axonal injury during the course of this rare malformation.
